# The Development and Characterization of Biobased Film Formulations Made of Chitosan, Gelatine, and Gum Arabic with the Addition of Lemon Balm (*Melissa officinalis* L.) Extract as a Novel Food Packaging

**DOI:** 10.3390/molecules31101582

**Published:** 2026-05-09

**Authors:** Mia Kurek, Ana Soldo, Petra Babić, Nasreddine Benbettaieb, Frédéric Debeaufort, Tea Sokač Cvetnić

**Affiliations:** 1 Laboratory for Food Packaging, Faculty of Food Technology and Biotechnology, University of Zagreb, Pierotti Street 6, 10000 Zagreb, Croatia; mkurek@pbf.hr (M.K.); asoldo@pbf.hr (A.S.); ppisonic@pbf.hr (P.B.); 2 PCAV Team, Joint Unit PAM, Food Processing and Microbiology, National Research Institute for Agriculture, Food and Environment (INRAé), Institut AgroDijon, University Burgundy Europe, 1 Esplanade Erasme, 21000 Dijon, France; nasreddine.benbettaieb@iut-dijon.u-bourgogne.fr (N.B.); frederic.debeaufort@u-bourgogne.fr (F.D.); 3 BioEngineering Department, IUT-Dijon, University Burgundy Europe, 7 Blvd Docteur Petitjean, BP17867, CEDEX, 21078 Dijon, France

**Keywords:** edible film, chitosan, gelatine, gum arabic, lemon balm extract, bioactive compounds

## Abstract

The aim of this study was to use lemon balm extract (*Melissa officinalis* L.), prepared via microwave-assisted extraction, for the development of novel formulations of functional edible films based on chitosan, gum arabic, and gelatine (simple and blended formulations). This study focused on changes in the antioxidant properties of enriched films, in addition to their physicochemical and barrier performance for potential applications. Thickness, colour, transparency, water solubility, gas and water vapour permeability, total polyphenol content, and antioxidant capacity were evaluated. The addition of lemon balm extract resulted in an increased polyphenol content (of about 30%) and enhanced antioxidant properties (approximately three-fold), without influencing hydration-related properties (solubility, moisture content and water absorption). These parameters were significantly influenced by the matrix structure (neat chitosan vs. blends with gelatine and gum arabic). Significant increases in the oxygen (three-fold for neat chitosan and five-fold for blends) and carbon dioxide (21-fold for blends) permeability coefficients were also observed in all films with extracts. However, all values remained below 30 × 10^−5^ cm^3^ m^−1^ d^−1^ bar^−1^, indicating that all films retained good gas barrier properties. The results indicate the potential of the developed material for applications in active food packaging as a sustainable alternative to traditional packaging materials, which should be further validated through studies on real food systems and shelf-life evaluation.

## 1. Introduction

As the principal function of food packaging is food product protection, specifically from external physical, chemical, and biological agents, synthetic polymers predominate in its production. The world generates an annual output of 367 million tonnes of plastic packaging, representing a significant source of environmental pollution [1].

In recent years, research efforts have been directed toward the development of alternatives to conventional plastic packaging materials, primarily driven by increasing consumer awareness of personal health, food and nutritional quality, food safety and environmental sustainability. Consequently, innovations in the development of edible packaging films have led to the emergence of environmentally friendly, non-toxic, and biodegradable materials [2,3]. Films made of edible materials are thin layers that act as a protective barrier for food and can be consumed in conjunction with it [4]. Polysaccharides, proteins, and lipids are some of the edible materials used (cellulose and its derivatives, starch, chitosan, alginate, and gums) [1,5]. Chitosan, gum arabic, and gelatine are all biodegradable, biocompatible polymers that are commonly combined to create versatile, non-toxic materials used in different fields, like regenerative medicine, therapeutic transport, controlled release encapsulates, and food packaging. They form strong electrostatic complexes that create specially designed matrixes that can serve as carriers of functional compounds for tailored release in packaging films used for the extension of food shelf life.

Chitosan has often been recognized as a high-potential biomaterial for replacing synthetic polymers [6,7]. Due to the dense structure created by linking (1,4)-linked 2-amino-2-deoxy-β-D-glucan units [7,8], it forms a good water vapour barrier when compared, for example, to starch- or paper-based materials, but its gas barrier efficiency still strongly depends on the environmental RH. When tested and used in dry environments, it shows great barrier protection against gases, representing its main advantage over other polymers. Valued for its biodegradability, environmental friendliness, and low cost, it is nevertheless limited by its low mechanical strength, restricted elasticity, brittleness, and water sensitivity [3,9]. Gelatine is obtained from the hydrolytic degradation of collagen protein, providing numerous benefits due to its distinctive and reactive structure [8,10]. Although, in dry conditions, it provides a great barrier to gases, this ability degrades in “real product” applications with higher water activity due to its hydrophilicity [8]. Gum arabic (also known as acacia gum), an amphiphilic polysaccharide, ranks among the most established and recognized natural gums used in the food, beverage, pharmaceutical, textile and cosmetic sectors [4,11]. Its application to the food packaging sector has not been well elaborated, with only a few studies on its use in edible coatings [11]. Over the past 10 years, more than 4000 and 1600 studies on chitosan and gelatine monolayers, respectively, have been indexed in the WoS; however, their combination remains insufficiently studied.

Combining polymers (e.g., polysaccharides, proteins, and lipids) creates superior, durable, and customized packaging solutions. Since chitosan is a unique biopolymer with cationic properties, owing to the cationic amine groups (-NH_2_) that become protonated amino groups (-NH_3_^+^) in acidic conditions, it is a great candidate for combination with anionic polymers (e.g., alginate, carrageenan, and gelatine) that could result in the creation of polyelectrolyte complexes. Therefore, considering the above-mentioned drawbacks of chitosan and gelatine, blending these two biopolymers through hydrogen bonding could create complexes with excellent physical properties at an adequate pH that could provide effective food protection during transportation and extend the shelf life of perishable products, especially those sensitive to oxygen, light, and temperature variations [3,12,13]. In the same manner, chitosan and gum arabic could form blends through the strong electrostatic interactions between the amino groups of chitosan and the carboxyl groups of gum arabic and serve as food packaging [14]. Due to its amphiphilic character, adding gum arabic to chitosan, in combination with other compounds, could provide interesting functional properties.

Chitosan–gelatine and chitosan–gum arabic complexation could be enhanced by incorporating plant-based bioactive compounds into ternary complexes, which could be further enhanced by providing additional functionalities, such as antibacterial properties and oxidation resistance [14,15]. Numerous research studies have indicated that adding different plant-based bioactive components to biopolymer films can significantly improve food packaging materials. This enhancement is achieved by boosting their functionality through antimicrobial, antioxidant, and anti-enzymatic properties [1,16,17,18]. Several undesirable effects of synthetic compounds on human health are forcing the food industry to look for alternative solutions in response to consumer pressure. Lemon balm (*Melissa officinalis* L.) has gained the interest of researchers due to its significant amount of natural antioxidants [19]. It is a medicinal herb used as a strong antimicrobial preservative [20,21,22], rich in a variety of volatile compounds (geranial, geraniol and citronellol), phenolic compounds (gallic acid, rosmarinic acid, caffeic acid, and porotocatehuic acid) and flavonoids (quercetin, luteolin, rutin, and catechin) [20,21,23]. The production and extraction of natural ingredients from plants can be achieved through various techniques. Nowadays, the accent is on those that eliminate the need for organic solvents and reduce energy consumption and, at the same time, maintain the naturality of the produced extract. Among these, microwave-assisted extraction has emerged as a green extraction technique due to the short extraction cycles, reduced solvent amounts, high extraction rates, and lower costs. The benefits of microwave-assisted extraction (MAE) for the preparation of lemon balm extracts for further use are shown in a previous study [20].

In earlier research, authors studied active films made of lemon balm essential oils with carboxymethyl chitosan and locust bean gum [24] and pectin/carrageenan [25]. Tayebi et al. [26] used *Melissa officinalis* for the development of photo-cross-linked nanofibers. Nanofibers were produced by electrospinning, emphasizing a key role of lemon balm as a natural bioactive component in active food packaging. Incorporation of the plant extract significantly enhanced mechanical strength and water resistance as well as the functional performance of the material, making it more effective in extending food shelf life. Overall, the study highlighted the importance of *Melissa officinalis* as a natural, sustainable additive that enabled the development of eco-friendly packaging. Also, the effect of lemon balm oil incorporated in gelatine edible film on tofu quality during storage was investigated by Riyad et al. [22]. Results have shown that edible coating with lemon balm oil slowed down microbial growth, which extended the shelf life of tofu up to 12 days. However, to our knowledge, very few studies are available on the incorporation of lemon balm water extracts.

It can also be hypothesized that chitosan could react with lemon balm via the Schiff base and interact with gelatine and/or gum arabic via electrostatic attraction forces. Further, the emulsification properties of chitosan in complexes could help chitosan to react with more lemon balm, thus resulting in better film functionality.

Despite the extensive research on chitosan, no studies have specifically addressed the development and characterization of chitosan, gelatine, and gum arabic-based formulations incorporating lemon balm water extract for food packaging applications. In this context, the present study examines the influence of two different anionic polymers, gelatine and gum arabic, on the complexation behaviour and resulting film properties of chitosan-based films. Furthermore, this study aims at evaluating whether blending the selected polymers can improve stabilization of the complex, while also improving the effective encapsulation, providing better protection during storage, and enabling controlled release. Further, the impacts of lemon balm extract, freshly prepared via microwave-assisted extraction, on the physicochemical (colour, transparency, thickness, water content, solubility), barrier (permeability to oxygen, carbon dioxide and water vapour) and antioxidant (total phenols, antioxidant activity and DPPH inhibition) properties of novel film formulations, as a potential packaging for fish, were studied.

## 2. Results and Discussion

### 2.1. pH of Biopolymer Film Dispersions

**Figure 1:**
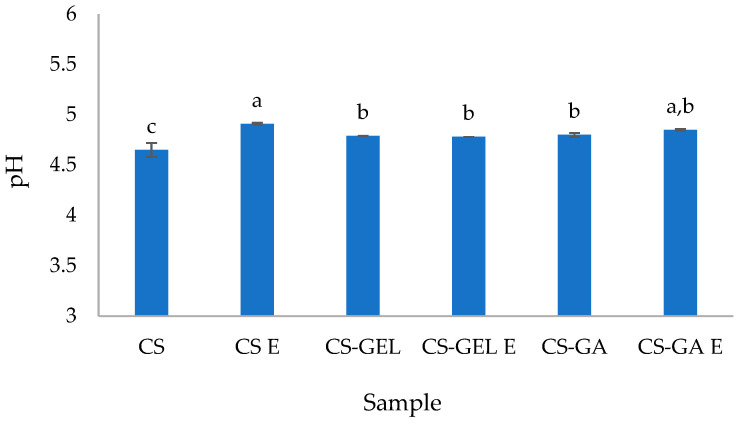
pH value of prepared biopolymer film dispersions (^a–c^ different lowercase superscript letters denote significant differences (*p* < 0.05) between values obtained for different film sample formulations).

The pH values (Figure 1) of all film-forming dispersions ranged from 4.65 to 4.91, indicating acidic conditions. This was expected due to the preparation of chitosan solutions in an aqueous acetic acid. In acidic media, the amino groups of chitosan undergo protonation to become positively charged (protonated) amino groups (NH_3_^+^) of the glucosamine units, thus making chitosan a polycationic polymer. This protonation and positively charged chitosan with free cationic sites on the polymer backbone enable the electrostatic interactions with negatively charged functional groups (e.g., carboxylate groups) present in gelatine and gum arabic, as well as hydrogen bonding within and between polymer chains. Such interactions contribute to the formation of a better-organized polymer network and are strongly dependent on the pH of the system, as pH controls the degree of ionization of all biopolymers involved. The neat chitosan dispersion had the lowest pH, which increased after the addition of gelatine and gum arabic, with no significant differences between them. This increase can be explained by partial neutralization effects and the buffering capacity of gelatine and gum arabic, which contain both acidic (–COOH) and basic (–NH_2_) functional groups. In particular, gelatine has carboxyl groups that can interact with protonated chitosan, reducing the concentration of free protons in solution and leading to a slight increase in measured pH (Figure 1). However, the polymer ratios are also important, as discussed hereafter. The interactions between gelatine and chitosan were influenced by both the initial pH of the individual polymer solutions and the final pH after mixing. Gelatine was initially prepared at pH 5.4, where it carries a net negative charge due to being above its isoelectric point, while chitosan was prepared at pH 4.6, where its amino groups were highly protonated, resulting in a strong positive charge. Upon mixing, the system was equilibrated to a final pH of 4.8. Under these conditions, oppositely charged macromolecules interact through electrostatic attraction. Although the charge density at this pH is lower than at higher pH values, it is still sufficient to promote the association of chitosan with negatively charged sites in gelatine. These interactions may also have been reinforced by hydrogen bonding and hydrophobic contributions from both biopolymers, potentially leading to the formation of an interpenetrated polymer network structure, while still involving extensive association between polymers. Consequently, the formation of some heterogenous aggregates was also expected. As mentioned earlier, polymer ratio plays an important role in controlling the extent of electrostatic neutralization and the properties of the resulting polymer matrix. In both systems, chitosan was present in excess; however, the nature and charge density of the anionic polymer (gelatine or gum arabic) in the blending dispersion determined the efficiency of complex formation. In the case of gelatine (2:1 chitosan/gelatine), the limited availability of negative charges near its isoelectric point possibly may have restricted complete charge compensation, resulting in partially formed complexes. In contrast, for gum arabic formulations (2:0.2 chitosan/gum arabic), despite its lower concentration, gum arabic that is rich with anionic sites acts mainly as a crosslinking agent and provides a higher density of anionic sites. In both mixtures, the resulting dispersions were dominated by chitosan in the formed polyelectrolyte systems, which were stabilized by strong ionic interactions and secondary bonding forces, with the final pH remaining below 5 for all blends. The addition of lemon balm extracts did not result in statistically significant pH shifts in most formulations, except for the neat chitosan dispersion. Since the extract contains only low concentrations of acidic or basic compounds, as confirmed by its compositional characterization (Section 2.2.4), its direct contribution to pH changes is expected to be minimal. The slight increase in pH observed, particularly in the neat chitosan dissolution, can, therefore, be mainly attributed to dilution effects resulting from the incorporation of the aqueous extract (at 10% *v*/*v*) rather than to the intrinsic acidity or basicity of the extract constituents. The pH of the film-forming dispersions is an important parameter, as it indicates whether the system is acidic or base, which in turn can influence biopolymer interactions, particularly protein charge, polysaccharide behaviour, and the stability of incorporated active compounds. The observed pH variations may arise from differences in the intrinsic acid–base properties of the added components, as well as from potential interactions between biopolymers and active compounds in the dispersion. These factors can affect the organization of polymer chains and the properties of the resulting dry films. A similar pH behaviour for chitosan–gelatine complexes was reported by Kurek et al. [3]. During complex formation, the highly charged chitosan causes additional ionization of gelatine carboxyl groups [27].

### 2.2. Biopolymer Film Characterization

#### 2.2.1. Moisture Content, Solubility and Water Absorption

Table 1 shows the moisture content (*M*), solubility (*S*) and water absorption (*WA*) of the studied films. The highest moisture content was obtained for CS E. The moisture content of CS-GEL was in accordance with the literature data [3,27]. The addition of the extract did not influence the moisture content or solubility of the blended films containing gelatine or gum Arabic; however, it led to an increased moisture content in chitosan films (single polymer). This effect can be attributed to the presence of the plasticizer and its different effect on the chitosan polymer structure. In addition, this effect was not observed in CS–GEL or CS–GA films, likely due to their inherently higher hydrophilicity. Additionally, heating during film formation at elevated temperatures may have led to thermal crosslinking of gelatine or arabic gum, thereby impacting their solubility. When glycerol is incorporated into the chitosan matrix, it interacts with the polymer chains through hydrogen bonding with the hydroxyl (–OH) and amino (–NH_2_) groups of chitosan. It also acts as a hygroscopic compound, exhibiting a strong tendency to attract and retain water molecules, thereby increasing the overall water affinity of the system. Its plasticizing effect leads to a loosening of the polymer structure, which is consequently associated with increased water vapour transfer rates, as discussed later in this study. Plant water extracts and their polyphenolic compounds can also interact with chitosan amino groups or break down its semi-crystalline structure (hydrogen bonds), leading to an enhanced ability to bind water [28]. Furthermore, as the extract was water-based, its addition further promoted chitosan hydration [29]. In contrast, when gelatine or gum arabic were linked to chitosan, hydration decreased, most likely due to the formation of polyelectrolyte complexes between oppositely charged biopolymers. These electrostatic interactions promote the formation of an intermolecular network that limits the mobility of polymer chains and reduces the overall water uptake compared to neat chitosan films. However, while no significant differences in film solubility were observed in CS-GEL formulations, CS-GA blends exhibited significantly higher water absorption and swelling. Even though a reduction in swelling capacity could be expected in CS-GA blends due to electrostatic interactions between oppositely charged polymers, the increased water absorption was attributed to the highly hydrophilic and branched structure of gum arabic, which is capable of absorbing substantial amounts of water even after the formation of polymer network and ionic association with chitosan. In addition, the hygroscopic nature of glycerol further contributed to water retention by increasing the free volume and enhancing polymer–water interactions within the matrix. This was also consistent with permeation results, which suggested the formation of a more crosslinked-like structure in CS–GA films.

High water solubility and water absorption may indicate a greater affinity for water. While this characteristic can be advantageous for some applications, it can adversely impact film integrity and barrier performance. Films with high water solubility are more prone to rapid degradation, but they may also be easily modified to improve physical and chemical properties [30].

A higher solubility value for chitosan film (50.35 ± 0.78%) has been reported by Jridi et al. [31]. The authors also observed that blending gelatine with chitosan reduced the solubility of the resulting films [31]. Solubility values reported in the literature for various polymers vary. For example, Kurek et al. [3], Leon-Lopez et al. [1] and Alnadari et al. [32] found that the presence of gallic acid, orange essential oil, pear extracts, or *Cinnamomum camphora* fruit peel waste decreased the solubility of chitosan–gelatine, gelatine, and chitosan–gum arabic films, respectively. This was probably due to a denser matrix structure, leading to reduced polymer–water interactions and bounded water content (CS (1502.68 kg/dm^3^) vs. CS E (1093.03 kg/dm^3^); CS-GEL (556.17 kg/dm^3^) vs. CS-GEL E (542.36 kg/dm^3^); CS-GA (1147.99 kg/dm^3^) vs. CS-GA E (730.16 kg/dm^3^)). In relation to the previously observed pH behaviour of the film-forming dispersions, the observed differences in dry film density could also be attributed to variations in the extent of the formation of polyelectrolyte complexes and the resulting network compactness. Chitosan had the highest density because of the strong chitosan self-association during drying. Under stable acidic conditions, chitosan polymer chains form extensive hydrogen bonding and ionic clustering as water evaporates. The addition of extract led to a decrease in film density, which was attributed to the more residual water associated with the extract and, consequently, to a less ordered packing of polymer chains within the crystalline structure. In CS-GA, strong electrostatic interactions between chitosan and gum arabic promoted the formation of densely packed ionic complexes, reducing interactions of polymer chains with water and, thus, resulting in both lower moisture content and higher dry density after film formation. In contrast, in CS-GEL films, in polymer dispersion, less effective charge-driven structures at mixed pH conditions limited the formation of compact polyelectrolyte structures. This led to more open dry matrices with reduced packing efficiency and, consequently, reduced density. In contrast, some studies have shown that the incorporation of plant extracts increases the solubility and swelling capacity of chitosan-based films [28].

#### 2.2.2. Colour and Transparency

Optical properties, such as colour and transparency, are important indicators of biopolymer film quality, as they influence product appearance and, consequently, consumer acceptability [1]. A high L* value indicates a colourless, bright and uniform sample; positive a* values represent redness; negative values indicate green tones; and positive b* values correspond to yellowness [12]. Table 2 shows the optical properties of the prepared formulations.

As can be noticed, CS-GEL had a significantly higher L* value (90.46 ± 0.46), which decreased upon the addition of the extract, whereas the CS-GA E film was the darkest (69.79 ± 3.92). The addition of the extract led to an increase in a* values, rendering them positive compared with their corresponding non-enriched film formulations. The extract itself had a yellow/brown colour, with L*, a* and b* values of 39.75 ± 0.38, 1.33 ± 0.09, and 7.11 ± 0.08, respectively. Films with GA had more pronounced red hue, while the presence of the extract made films more yellow. All b* values were positive, with higher values observed for films with extracts. All blended formulations were significantly different from the neat chitosan film, demonstrating the strong influence of the extract on optical properties. The highest total colour difference was calculated for CS-GA E, resulting in colour changes also visible to the human eye (ΔE > 3) [33]. This was attributed to the combined influence of both gum arabic on redness and lemon balm extract on film yellowness. Thus, resulting films had increased brownish tones (Table 2). Similarly, Chan-Matu et al. [18] and Nxumalo et al. [28] reported a significant colour change of chitosan-based films upon the addition of plant extract.

**Figure 2:**
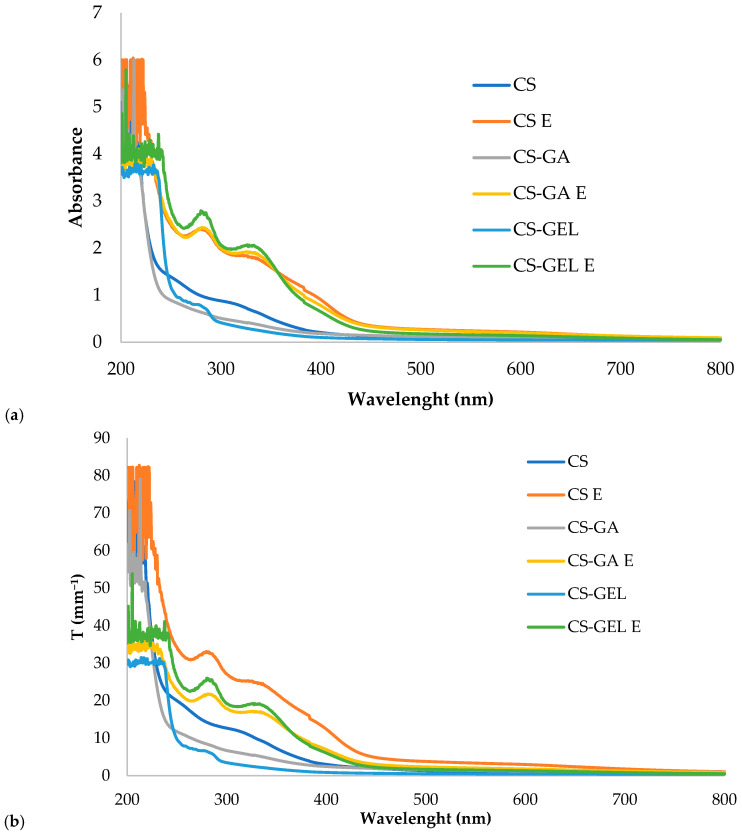
Measurement of the film transparency in the UV-VIS spectral range: (**a**) transparency absorbance values of tested films, and (**b**) transparency expressed as thickness-normalized results.

Transparency refers to the ability of a material to transmit light with lower values, indicating more transparent samples [34]. As shown in Table 2, lower values were obtained for films without the extract, meaning they were more transparent in comparison to ones with the extract. These results can be attributed to the presence of the polyphenolic compounds in the extract, which are known to absorb or block UV radiation [35]. The whole UV/VIS spectra of the films are given in Figure 2a, while thickness-normalized transparency values, T (mm^−1^), across the entire spectral range are presented in Figure 2b. Thickness normalization enables a more direct comparison of the optical properties of films with different thicknesses over the full wavelength range. Taking thicknesses into account, the differences among the films follow the same trends observed in absorbance measurements but became more pronounced, allowing clearer differentiation between film types. Films without extracts showed significantly lower absorbance in the whole measured spectral range, from 200 to 800 nm. In the UV range (below 400 nm), differences were even more significant than in the visible spectral range. The incorporation of lemon balm extract further enhanced the barrier properties, with no significant differences observed among the different biopolymer formulations. For UV-B light, blending chitosan with GEL and GA improved the barrier by an average of 65%, and this effect was further enhanced by the addition of the extract. In particular, increases of 262% and 280% were observed in CS–GEL and CS-GA films with extracts, respectively, whereas CS films with the extract exhibited an average improvement slightly above 250%. A similar trend was observed for UV-C light. Neat blends improved the barrier properties by approximately 200% and 240% when CS was combined with GEL and GA, respectively. The addition of lemon balm extract resulted in even greater enhancements, reaching approximately 360% for CS, 315% for CS–GEL, and 360% for CS–GA films. High-energy UV radiation, particularly in the UV-C (100–280 nm) and UV-B (280–315 nm) ranges, is known to induce nutrient degradation and negatively affect packaging integrity. Accordingly, the developed blends showed strong potential as packaging materials due to their effective UV-barrier properties. This functionality is especially important for fish packaging, as fresh fish is highly susceptible to ultraviolet exposure. Although UV-C radiation is widely applied for decontamination purposes, excessive or uncontrolled doses may induce oxidative deterioration in a dose-dependent manner [36,37]. Enriched films also show a good barrier in the violet/blue light range (400–470 nm) that is known to speed up chemical reactions, promote nutrient loss, induce lipid rancidity, and affect the sensory quality of food. Visible light exposure during fish storage plays a critical role in reducing quality by accelerating lipid oxidation, causing colour degradation, and reducing shelf life [38,39]. Similar results were also reported by Chan-Matu et al. [18] who investigated different percentages of *Moringa oleifera* ethanolic extract added to a chitosan-based film and observed that higher extract contents resulted in lower transparency values. Furthermore, the CS-GEL film had the lowest transparency, similarly to previous studies by Kurek et al. [3] and Jridi et al. [31]. The CS film had higher opacity values (1.09 ± 0.11 mm^−1^) compared to the CS-GEL film (0.68 ± 0.07 mm^−1^). The lower opacity observed in blended films was possibly due to the loss of anisotropy, which led to a decrease in light scattering in ordered domains in the CS matrix. Chitosan polymer chains form ordered regions that can scatter light and, thus, make films less transparent. Adding gelatine increased the amorphous fraction in the film that scatters less light, so the film appeared more transparent. Also, chitosan and gelatine can form interpenetrating polymer networks (IPNs), and in a homogeneous IPN, the polymer domains are finer and more uniform, minimizing phase separation. This uniform structure reduces light scattering, allowing light to pass more easily and making the film more transparent. In enriched films, plant compounds interact with chitosan chains via hydrogen bonding or electrostatic interactions, creating micro-phase separation. These heterogeneous regions scatter light, further reducing transparency. In addition, pigments from plants also absorb visible light, as explained earlier.

#### 2.2.3. Thickness and Permeability to Oxygen, Carbon Dioxide and Water Vapour

Film thickness is an important parameter, as it directly influences mechanical properties and transparency, and can consequently affect the quality and shelf life of food products [1]. Moreover, barrier properties are key parameters for evaluating the performance of packaging films during food preservation, including oxygen, water vapour, and light barrier properties. These characteristics are closely associated with oxidation processes, microbial ingress and growth, and overall food spoilage [12]. The results of thickness and barrier properties for the tested films are presented in Table 3.

The thickness of the films ranged from 69.50 ± 20.02 to 120.50 ± 37.71 µm. Interactions between chitosan and other biopolymers, as well as the incorporation of lemon balm extract, resulted in significant variations in film thickness. Also, an increase in solid content in the films could cause structural changes, increasing the thickness values [1]. In other words, the thickness of chitosan-based films is related to their nature and composition [18].

Water vapour barrier property is a vital feature, limiting the water vapour’s ability to pass the film. It is an important parameter for evaluating the material efficiency in protecting food against spoilage [12]. The water vapour barrier properties are expressed as the water vapour permeability (*WVP*) and water vapour transmission rate (*WVTR*). To maintain the freshness of food products, by inhibiting spoilage and microbial growth, WVP and WVTR should be as low as possible [28,30,40].

As shown in Table 3, complexation of chitosan with gelatine and arabic gum led to a slight decrease in water vapour permeability values. This is in line with the results on the film moisture content, as given in Table 1. To understand how film hydration at tested RH influenced the structure and, therefore, the organization of polymer chains on the macromolecular level, in further studies, it would be interesting to check water vapour sorption rates. This could elucidate the critical water content on the film surface as well as the possible water clustering and differences in diffusion. The addition of extracts did not influence the permeation of water vapour, indicating that even though the hydrophilic character of films was increased, complexes that were formed were not influenced by the components from the extracts, or in other words, the network mesh remained sufficiently organized and not modified by extracts.

Contrarily to the present study, Leon-Lopez et al. [1] noticed an increase in water vapour permeability following the incorporation of various hydroalcoholic extracts, attributing this effect to the presence of phenolic compounds that altered the biopolymer structure and, consequently, WVP. Although, the lemon balm extract used in the present study also contains various polyphenolic compounds, as given in an earlier study [20], these compounds may form hydrogen and even covalent bonds with polar groups of biopolymers that remain free for bonding after complexation in polyelectrolyte complexes. Such interactions can limit the availability of hydrogen groups for hydrophilic bonding with water, thereby reducing film hydrophilicity and overall water affinity [30]. In CS-GA formulations, the lower water vapour permeability values could also be partially attributed to the reduced plasticizer content (10% in comparison to 20% in other formulations). As discussed earlier, the CS-GA system had a more crosslinked structure, while sorption of water in films is led by the hygroscopicity of glycerol. However, in this case, glycerol did not impact the water vapour permeation. The presence of plasticizers in biopolymer film formulations could influence the increased water binding and higher diffusivity rates, due to the entanglement of polymer chains in the presence of plasticizer.

**Figure 3:**
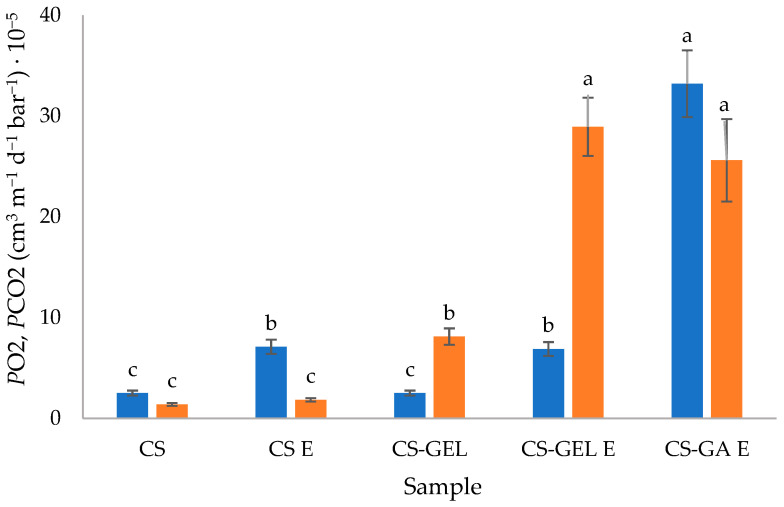
Gas permeability coefficients for oxygen and carbon dioxide of prepared biopolymer films (■ oxygen permeability coefficient *P*O_2_; ■ carbon dioxide permeability coefficient *P*CO_2_) (^a–c^ different lowercase superscript letters within a column denote significant differences (*p* < 0.05) between values obtained for different biopolymer formulations). CS-GA—not measured due to the poor mechanical properties—films did not manage the applied measuring method and were broken during analysis due to the strong differences in partial pressures.

Considering the results (Figure 3) for gas permeability coefficients for oxygen and carbon dioxide, there was a significant difference between the formulations, with no evident trend. Films with GA were significantly more permeable to O_2_ and CO_2_ than other film formulations, probably due to the lack of density (values given in Section 2.2.1). The addition of extracts led to an increase in permeability values, with more significant differences in complex formulations. This could be related to the higher moisture content and solubility of those films in water (Table 1). Gas permeation in polymers is determined by the diffusivity and solubility of gases within the polymer matrix. The higher moisture content and increased water solubility of the enriched polymers, therefore, facilitate gas permeation. In addition, the density of the IPN network also affects this behaviour. The increase was more important for CO_2_ than for O_2_. Two possible explanations can be proposed. First, the antioxidant character of lemon balm extract may have partially restricted the diffusion of oxygen through the film (oxidation occurred). Second, the higher CO_2_ permeability observed in CS-GEL films could also be related to the higher solubility of CO_2_ in glycerol. Even though CO_2_ molecules are slightly larger than O_2_ molecules, gas solubility in glycerol is governed more by molecular interactions than by size. Therefore, in glycerol-plasticized biopolymer films (like gelatine or chitosan), CO_2_ tends to dissolve and permeate more easily than O_2_. This is why many biodegradable packaging films show higher CO_2_ permeability than O_2_ permeability [41]. Even though comparison with the literature data is difficult since no same exact formulation could be found, the values were still in the same order of magnitude as given for chitosan and CS-GEL films in previous studies [3].

#### 2.2.4. Mechanical Properties

**Figure 4:**
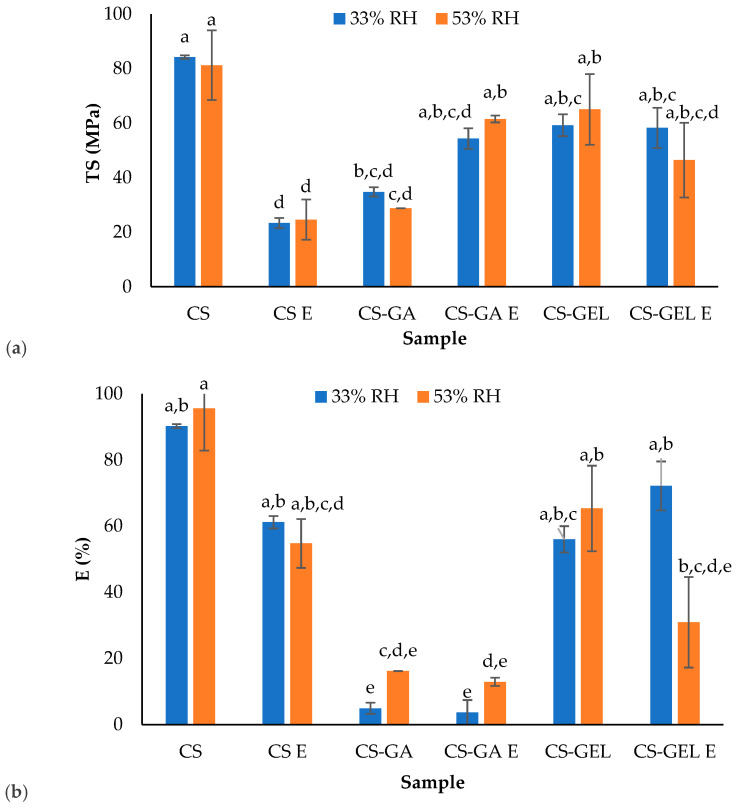

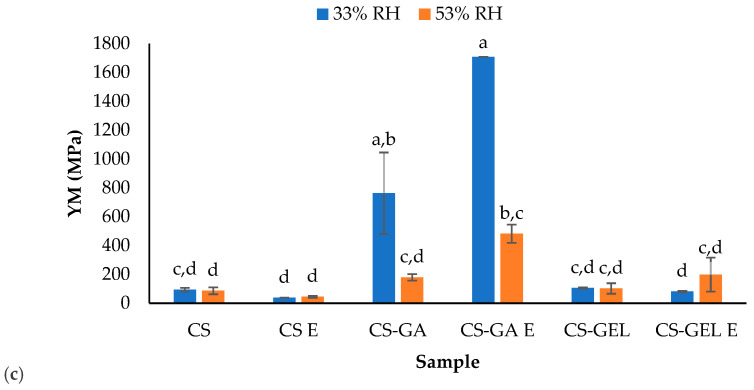
Results of film mechanical properties: (**a**) tensile strength, (**b**) elongation and (**c**) Young modulus (^a–e^ different lowercase superscript letters within a column denote significant differences (*p* < 0.05) between values obtained for different biopolymer formulations).

The mechanical properties of the films were tested for films equilibrated at two different relative humidities: 33% and 53% RH (Figure 4). This approach was used because hydrophilic biopolymer films are highly sensitive to water content (Section Moisture (M), Solubility in Water (S) and Water Absorption Capacity (WA)) which acts as a plasticiser and significantly affects tensile properties. Testing at different RH levels allows for direct assessment of how moisture modifies tensile strength, elongation, and Young’s modulus, providing insight into the plasticizing effect of water within the polymer matrix. Specifically, higher RH conditions are expected to increase chain mobility and flexibility, while lower RH conditions result in stiffer and more brittle films. Results from the present study showed that the mechanical properties of the films were more influenced by composition rather than relative humidity (RH). CS films had high tensile strength and relatively stable Young’s modulus at both RH levels, indicating the formation of a dense and organised polymer network. Only minor changes were observed with increasing RH, suggesting limited susceptibility to moisture-induced plasticization compared to blended systems. In contrast, with the addition of extract, in CS E films, significantly lower tensile strength and Young’s modulus compared to CS were observed. This indicates that the enriched formulation was more flexible. For these films, there were also no significant changes in mechanical properties with RH, indicating that their mechanical behaviour was primarily influenced by composition rather than variations in environmental moisture. In contrast to neat CS, CS-GA blends showed stronger sensitivity when stored at different RH. At 33% RH, high stiffness (high Young’s modulus) was measured and very low elongation, indicating a brittle and highly rigid structure. However, at 53% RH, a strong reduction in stiffness occurred, while elongation was increased. This behaviour confirms that gum arabic introduces a highly moisture-responsive network in which water severely disrupts intermolecular interactions and influences plasticization, as observed in mechanical tests. The addition of extract did not significantly influence the mechanical properties of CS-GA blends. For CS-GEL blends, no significant changes in all measured parameters were observed, while films with extract showed decreased elongation at higher RH. Comparing all formulations, surprisingly, the most elastic films were CS films, followed by CS-GEL and CS-GA blends.

#### 2.2.5. Total Phenol Content and Antioxidant Activity

**Figure 5:**
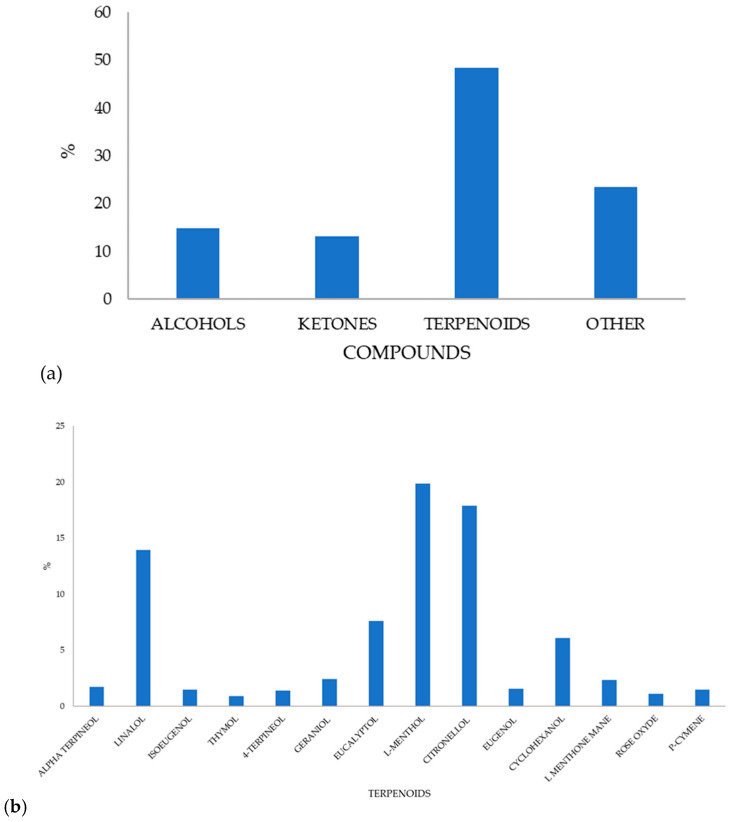
Content of volatile compounds (**a**) and terpenoids (**b**) in lemon balm extract, expressed as the percentage (%) of total terpenoids detected. Compound identification is adapted from Babić et al. [22], while the quantitative data (percentages of individual terpenoids) were analysed in this study. All the experiments were conducted in triplicate.

The development of active biopolymer packaging includes improving aspects such as food protection [18]. The application of antioxidants is a promising technology for extending the shelf life of food products and plays a crucial role in determining the effectiveness of active films, as it indicates their ability to prevent the oxidation of treated food products [30,42]. Upon application, these films could also release active compounds (e.g., natural extracts, polyphenols) directly onto food surfaces and consequently minimize rancidity, maintain sensory quality (colour, flavour), and improve nutritional value. Since the activity is attributed to the content of components having a free electron to donate, when mixed and entrapped in biopolymer films, it is important to check this activity. In the present study, lemon balm extract prepared via microwave-assisted extraction was tested for its polyphenolic profile, total phenolic content, content of chlorophylls, total flavonoids, and antioxidant activity. The principal results are given in a separate study published recently. Most of the detected volatiles belonged to the terpenoid class, followed by alcohols, ketones, and smaller proportions of other compounds (acetates, sulphides, acids) (Figure 5a). The relative proportions of the main volatile terpenoids are given in Figure 4b. The profile of present terpenoids also contributes to the colour of both the extract and film, as discussed earlier, thereby influencing film transparency and light scattering. Even though major compounds, such as eucalyptol, L-menthol, eugenol, citronellol, and linalool, are largely colourless, the yellow–brownish colour of the film likely arises from other factors, like oxidation and temperature changes during film drying. During film casting and drying, volatile terpenoids can oxidize or react with chitosan to form quinone-like compounds, resulting in the yellow-brown colour. In addition, chitosan contains amino groups that can interact with phenolics or eugenol, and these interactions produce slightly coloured complexes, which are brownish or yellowish. The extract was significantly richest in rosmarinic acid (2472 mg/L), followed by ferulic acid (117 mg/L), naringin (111 mg/L), quercetin 3 glucoside (69 mg/L) and vanillic acid (58 mg/L), while protocatechuic, chlorogenic and caffeomalic acid were lower than 25 mg/L, oleuropein, kaempferol and quercetin around 10 mg/L [20]. Its total phenolic content measured using the Folin-Ciocalteu method was around 1126 mg_GAE_/L.

**Figure 6:**
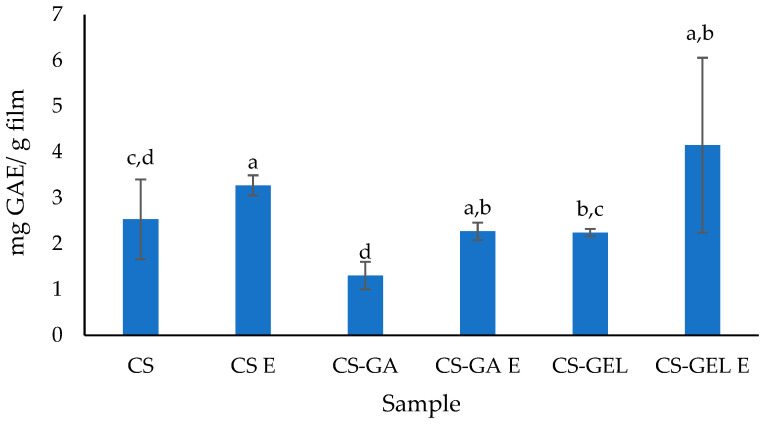
Total polyphenol content in biopolymer films released in water after 24 h (^a–d^ different lowercase superscript letters within a column denote significant differences (*p* < 0.05) between values obtained for different biopolymer formulations).

The results for total phenol content and antioxidant activity of the biopolymer films are shown in Figure 6 and in Table 4. Expressed results are related to the compounds released into water after 24 h. Due to the binding of molecules to the polymer matrix, as well as differences in film solubility and water absorption discussed previously, these values should be interpreted with caution, as the complete release of all active compounds may not have occurred under the experimental conditions. However, this method is common in the literature for expressing TPC in film materials. Although the biopolymer films without extract (CS, CS-GEL and CS-GA) do not inherently contain phenolic compounds, measurable TPC and antioxidant activity were measured. This may be attributed to interactions between FC reagent and amino groups of chitosan or certain amino acid residues present in gelatine [43]. It is also possible that phenols were partly bonded to polymer chains, hiding their possibility to react with the FC reagent, and, therefore, not showing significantly different results. The existing antioxidant activity of chitosan and gelatine films was also proved by Nxumalo et al. [28] and Leon-Lopez et al. [1].

The total phenol content was higher in biopolymer films with lemon balm extracts than in films without extracts, which was expected (Figure 6). The highest content can be noticed in CS-GEL E (4.25 mg_GAE_/g of film). Compared to the pure extract (around 1000 mg_GAE_/L) [20], the TPC values of films were significantly lower. However, considering that the extract concentration was 10% (*v*/*v*), it is to be expected that TPC in films should be lower, and the measured values correspond to these assumptions. In addition, the experimental setup must also be considered. Since films were immersed in water for 24 h, equal to time when release occurred, it is possible that not all the actives were released during this time. Theoretically, there should be a direct connection between how much water the film takes up (hydration) and how fast and how many hydrophilic phenols are released [44]. Also, it cannot be considered just as simple as more water equals more release, since the polymer structure matters a lot. When films were immersed in water, they progressively absorb it. This is evident from Figure 1. However, the total phenolic content and antioxidant activity measured in the release medium are not only governed by film hydration but are also influenced by structural changes within the polymer network and by potential interactions between the polymers and phenolic compounds. If hydration was the only determining factor, a higher release of phenolic compounds could be expected from CS-GEL E and CS-GA E films compared to the neat chitosan, due their higher WA values. However, even though CS-GA swells a lot, it also has many hydroxyl and carboxyl groups that can bond with phenols, resulting in reduced diffusion in the water and consequently lower TPC results. In the case of CS-GA E blends, even though gelatine also reacts with phenols, these reactions are possibly weaker or reversible, resulting in higher release. Interestingly, antioxidant activity determined by the DPPH assay was highest for CS-GA E films, indicating that this method reflects not only the quantity but also the reactivity of released compounds. Even small amounts of highly active phenols can give strong signals, therefore improving the overall result on the antioxidant activity.

The results of the antioxidant activity (Table 4) suggest that the addition of lemon balm extract resulted in films with the strongest DPPH inhibition. The highest antioxidant activity, as determined by both DPPH and FRAP assays, was observed for the chitosan-based film containing lemon balm extract. This enhanced activity was attributed to the presence of the active antioxidant compounds, as discussed earlier. In addition, the antioxidant activity was primarily associated with non-volatile compounds, principally rosmarinic acid, which exhibits significantly greater free radical scavenging capacity than volatile constituents like citronellol, linalool, or L-menthol [45,46]. The higher DPPH inhibition observed for the neat CS and CS–GA films compared to the CS–GEL film can be attributed to the greater availability of active functional groups that could donate hydrogen atoms or electrons to neutralize free radicals. In CS and CS–GA films, it is possible that there were more accessible amino groups in CS and phenolic groups (in the case of GA), which enhance radical scavenging activity. Indeed, it is known that gum arabic contains small amounts of phenolic compounds [47], which contribute to its antioxidant properties. In contrast, the incorporation of gelatine led to stronger intermolecular interactions (and even partial crosslinking) that reduced the availability of these active sites, thus resulting in lower DPPH inhibition. The results also indicate that the developed new film formulations serve as good protection (encapsulation) matrices for lemon balm aqueous extract, so even after processing and drying, films still have antioxidant activity that can be used efficiently for food applications. Also, the lowest values for DPPH inhibition and FRAP were obtained for the chitosan–gelatine film. Compared to results presented in the literature, lower values for antioxidant activity (DPPH inhibition and FRAP) for the CS-GEL film (5.85 ± 0.09% and 0.21 ± 0.05 mg AAE/g) were obtained [3].

## 3. Materials and Methods

### 3.1. Materials

Films were made using biopolymers chitosan (chitosan type 652, molecular weight 165 kDa, degree of deacetylation above 85%, France Chitin, Marseille, France), gelatine (Louis Francois, Collégien, France) and gum arabic (Enologica vason s.p.a., San Pietro in Carino, Verona, Italy). The vegetable glycerol (minimum purity 99.5%, E422, Dekorativna točka Ltd., Jakovlje, Croatia) was used as plasticizer. Lemon balm leaf (*Melissa officinalis* L.) for extract preparation was purchased from Suban Ltd. (Strmec Samoborski, Croatia). Distilled water and an aqueous solution of acetic acid (1% *v*/*v*, glacial acetic acid, J.T. Baker, Phillipsburg, NJ, USA) were used as solvents for film preparation. Magnesium nitrate hexahydrate (Sigma-Aldrich, St. Louis, MO, USA) was used in the preparation of a saturated solution to maintain relative humidity (53% RH).

Ethanol, sodium carbonate, sodium acetate trihydrate and iron (III) chloride hexahydrate were purchased from Kemika (Zagreb, Croatia). Folin-Ciocalteu reagent, 2,4,6-tris(2-pycrylhydrazyl) (TPTZ) and 2,2-diphenyl-2-picrylhydrazyl (DPPH) were purchased from Sigma-Aldrich Chemie (St. Louis, MO, USA). Hydrochloric acid was supplied by Carlo Erba Reagents S.A.S. (Val de Reuil, France). The standard of ascorbic acid was purchased from Fisher Chemical (Pittsburgh, PA, USA ). All chemicals used were of analytical reagent grade.

### 3.2. Methods

#### 3.2.1. Preparation of Lemon Balm Extract Using Microwave-Assisted Extraction

The dried lemon balm leaves were ground in a grinder (HR2860/55, Philips, Amsterdam, The Netherlands). Microwave-assisted extraction (Start S Microwave Labstation for Synthesis, Milestone, Sorisole, Italy) was conducted as follows: (a) solvent/water; (b) solvent/plant ratio—0.05 g plant/mL; (c) extraction time—10 min; (d) cycle power and temperature—500 W and 70 °C. Since, during extraction, the plant is mixed with the solvent, in order to separate active fraction, the sample was filtered (through 100% cellulose filter paper). Freshly prepared extracts were used for further characterization and film preparation.

The extract composition was characterised by HPLC for non-volatiles and volatiles with headspace solid-phase microextraction coupled with gas chromatography–mass spectrometry (HS-SPME/GC–MS), as described in Babić et al. [20].

#### 3.2.2. Preparation of Biopolymer Films

Films were prepared from 2% (*w*/*v*) chitosan dispersion (CS; dissolved in 1% (*v*/*v*) acetic acid), 1% (*w*/*v*) gelatine dispersion (GEL; prepared in distilled water) and 10% (w/pdm or 0.2 g/100 mL) gum arabic (GA; prepared in distilled water). The gelatine solution was heated to 60 °C for 20 min to ensure complete dissolution. Firstly, the individual polymer dispersions were mixed using a magnetic stirrer (Delab MS-H280-S4, Beijing, China) to achieve complete polymer dissolution. Subsequently, blend formulations were prepared by mixing the individual polymer dispersions according to the ratios specified in Table 5. Homogenization was performed using homogenizer (IKA Ultra turax T18, Staufen, Germany) at 12,000 rpm for 5 min, followed by continuous stirring in a magnetic stirrer at 600 rpm for 30 min. After homogenization, lemon balm extract (*Melissa officinalis*) (E) was added to the dispersions to obtain concentration of 10% (*v*/*v*). Afterwards, glycerol was added as a plasticizer at 20% (w/pdm relative to the polymer dry weight for chitosan and chitosan–gelatine formulations or 0.4 g/100 mL for CS, and 0.6/100 mL for CS-GEL) and 10% (w/pdm or 0.2 g/100 mL) for chitosan–gum arabic formulations. The lower glycerol content in formulations with gum arabic was selected due to the inherent plasticizing properties of gum arabic, as confirmed in preliminary laboratory trials. The final film-forming dispersions were stirred for additional 10 min at room temperature (23 ± 2 °C) to ensure homogeneity. For film casting, a defined volume (30 mL) was poured on a Petri dish (150 mm diameter) to ensure uniform thickness. The films were dried in a ventilated climatic chamber (Binder, KBF240, Binder Germany) for 24 h at 30 °C and 40% RH. After drying, the films were carefully peeled off the surface and conditioned in a desiccator at 53% RH and 23 ± 2 °C prior to further analysis. All analyses were performed at least in triplicate for each film type (batch).

#### 3.2.3. Determination of pH of Film Dispersions

The pH of film-forming dispersions was assessed with a laboratory FiveGo portable pH meter (Mettler Toledo, Greifensee, Switzerland). Measurements were made in triplicate, with results given as mean values ± standard deviation.

#### 3.2.4. Film Characterization

##### Moisture (M), Solubility in Water (S) and Water Absorption Capacity (WA)

The moisture content in the film, previously equilibrated to 33% RH, was calculated by subtracting the initial dry matter weight (*W*_i_) from the weight after drying (*W*_d_).

For assessing water solubility, the method outlined by Singh et al. [5] was followed, with some modifications [5]. Films were cut into 2 × 2 cm pieces, dried in an oven at 105 °C for 24 h, and weighted (*W*_1_). Each piece was then placed in a 50 mL beaker with 30 mL of distilled water, stored at 25 ± 1 °C for 24 h, dried again at 105 °C for another 24 h, cooled, and weighed to determine the mass of dry matter that remained undissolved in water (*W*_2_). The solubility of the films in water (S, %) was then calculated using the following equation (Equation (1)):

(1) $$S(\%)=\frac{W_{1}-W_{2}}{W_{1}}\times 100$$

The water absorption capacity of the film is considered according to its swelling capacity, also known as the swelling ratio, which was assessed using a standard method. [48]. The swelling capacity, after the immersion of the sample in the water for 24 h, was calculated using Equation (2):

(2) $$\;WA\left(\%\right)=\;\frac{W_{3}-W_{1}}{W_{1}}\times 100$$

where *W*_3_ is the mass of samples after swelling and *W*_1_ is the mass of the dry sample.

##### Film Thickness

The film thickness was measured by averaging 5 random points using a micrometre (Digimet, HP, Helios Preisser, Gammertingen, Germany) to an accuracy of 0.001 mm. The results are expressed as the mean values ± standard deviation.

##### Colour and Opacity

The colour was measured using colorimeter (Konica Minolta Spectrophotometer CM3500 d, Langenhagen, Germany). The device works on the principle of the CIE L*a*b* spatial colour diagram, where L* indicates lightness, a* reflects the spectrum from green to red, and b* covers from blue to yellow. The overall colour change was calculated using Equation (3):

(3) $$∆E=\sqrt{({L^{*}-L_{0}^{*})}^{2}+({a^{*}-a_{0}^{*})}^{2}+\;({b^{*}-b_{0}^{*})}^{2}}$$

The film without additives is considered as the reference, whose coordinates are represented by *L*_0_, *a*_0_, and *b*_0_, while *L*, *a*, and *b* denote the Hunter coordinates for the tested film sample. Measurements were conducted at ten different locations on the biopolymer film, with the results presented as mean values ± standard deviation.

The transparency of the developed biopolymer films was determined using absorbance measurements taken with a UV/VIS spectrometer (Lambda 25, PerkinElmer, Shelton, CT, USA) in the wavelength range from 200 to 800 nm. To calculate the film’s opacity value, Equation (4) was applied, where A_600_ represents the absorption at 600 nm and ‘x’ denotes the film thickness given in mm:

(4) $$Opacity\;value(T_{600})=\;\frac{A_{600}}{x}$$

##### Permeability to Water Vapour, Oxygen and Carbon Dioxide

The water vapour permeability of the films was assessed using a gravimetric method [49] that follows a modified standard approach tailored for edible materials [50], with a relative humidity (RH) differential of 75% (100 → 25%). Before taking measurements, the samples were kept in a desiccator under controlled humidity conditions at 33% RH. Film samples were cut into circular specimens (41 mm in diameter). The test cup was filled with 20 mL of distilled water, and the edge was sealed with vacuum grease, followed by the film sample, and a second Teflon ring secured with a lid. During handling, protective gloves were used. For each sample, for this analysis, measurements were performed in nine replicates. Water vapour permeability (*WVP*, g m^−1^ s^−1^ Pa^−1^) was calculated from the steady-state rate of mass loss using Equation (5):

(5) $$WVP=\;\frac{∆m}{∆t*A*∆p}*\;\chi$$

where Δm/Δt is the rate of moisture loss (g s^−1^); A is the exposed film area (9.08 × 10^−4^ m^2^); x is the film thickness (m); and Δp is the water vapour pressure difference across the film (Pa). Samples were subsequently stored in a climatic chamber (Binder KBF240, Tuttlingen, Germany) at 23 °C and 25% relative humidity. Mass changes were monitored using an analytical balance until a stable mass loss rate was achieved.

The findings are presented as water vapour permeability (*WVP*) and water vapour transmission rate (*WVTR*).

The permeability of the films to oxygen (O_2_) and carbon dioxide (CO_2_) was assessed using the manometric method with the GDP-C apparatus from Brugger Feinmechanik GmbH in Munich, Germany. The findings are presented as coefficients for oxygen and carbon dioxide permeability (PO_2_ and PCO_2,_ in cm^3^ m^−1^ day^−1^ Pa^−1^).

##### Determination of Mechanical Film Properties

The mechanical properties were determined using a texture analyser (TA.HDplus, Stable Micro Systems, Godalming, UK). Prior to measurements, the samples were stored in a desiccator under controlled humidity conditions (53% and 33% RH). Film samples with dimensions of 1 × 5 cm were clamped using grips (A/HDG, Stable Micro Systems, UK). Tensile testing was performed by extending the films at a speed of 50 mm min^−1^ until rupture. From the obtained data, the following parameters were determined: tensile strength (TS, MPa), Young’s modulus (YM, MPa), and elongation at break (E, %) (according to the ASTM standard method D882) [51]. *TS*, *YM*, and *E* were calculated from the stress–strain curves using the following equations:

(6) $$TS=\;\frac{F_{max}}{A_{i}}\;\;(MPa)$$

(7) $$E\;\left(\%\right)=\frac{l_{f}-l_{i}}{l_{i}}*100\;(MPa)$$

(8) $$YM=\frac{TS}{maximal\;deformation}\;\;\;\;\;(MPa)$$

with F_max_ maximum force at break, A_i_ initial cross-sectional area of the sample, l_i_ original length of the film between the grips (initial gauge length) and l_f_ final length of the film at the moment of rupture. Each film was tested in three replicates.

##### Determination of Total Phenol Content and Antioxidant Activity

Before the analysis of total phenolic content and antioxidant activity, the samples were prepared as follows: 0.2 g of dry biopolymer film was placed in vials with 20 mL of distilled water, and the solutions were stored at room temperature overnight to extract the active compounds. Next day, the solutions were filtered, and the supernatant was collected for analysis.

The total phenolic content (TPC) was assessed using a UV/VIS spectrometer (Lambda 25, Perkin Elmer, Waltham, MA, USA), following the procedure outlined by Shortle et al. [52]. Method relies on the colorimetric reaction of phenols with the Folin-Ciocalteu reagent. Concisely, 100 µL of the sample, 200 µL of Folin-Ciocalteu reagent, and 2 mL of deionized water were combined in glass tubes. After allowing the mixture to react for 3 min, 1 mL of a saturated sodium carbonate solution was introduced, followed by vortexing. The tubes were then placed in a water bath at 50 °C for 25 min. Once cooled, the absorbance was recorded at a wavelength of 765 nm. A calibration curve was established using gallic acid, and the results were expressed as mg of gallic acid equivalents (GAE) per gram of film. Each measurement was conducted in triplicate, and results are reported as mean values ± standard deviation.

The DPPH (1,1-diphenyl-2-picrylhydrazyl) method consists of reducing the DPPH radical in a methanol solution, following the procedure by Shortle et al. [51]. A precise amount of film samples, approximately 0.2 g, was placed in glass tubes with 0.004% DPPH solution for 30 min at room temperature in darkness. The interaction of DPPH radicals with antioxidants leads to the formation of stable molecules, resulting in a colour change from dark purple to pale yellow. The antioxidant activity of a sample is inversely related to the intensity of the DPPH’s blue-purple hue. The DPPH solution served as the control (A_control DPPH_). After the incubation period of 30 min, the absorbance was measured at a wavelength of λ = 517 nm. The percentage of DPPH inhibition was determined using Equation (9):

(9) $$\%inhibition=\frac{A_{control\;DPPH}-A_{sample}}{A_{control\;DPPH}}$$

The FRAP (Ferric-Reducing Antioxidant Power) consists of converting the colourless iron (III) tripyridyltriazine complex (Fe^3+^-TPTZ) into its vibrant blue ferro form (Fe^2+^), following the procedure Shortle et al. [52]. A calibration curve was prepared using ascorbic acid. The FRAP reagent was prepared by mixing 25 mL of acetate buffer (0.3 M), 2.5 mL of a TPTZ (2,4,6-tripyridyl-s-triazine) reagent, and 2.5 mL of iron (III) chloride in a ratio of 10:1:1. 300 μL of extracted film solution and 2250 μL of FRAP reagent was pipetted into glass tubes, vortexed, and incubated for 10 min at 30 °C in a water bath. After the reaction and cooling, the absorbance was measured at wavelength *λ* = 593 nm. The blank contained only the FRAP reagent and distilled water. The results are expressed as mg equivalent of ascorbic acid per gram of film. All measurements were performed in triplicate, and the results are presented as mean values ± standard deviation.

### 3.3. Statistical Analysis

Statistical analyses (one-way analysis of variance (ANOVA) and Tukey’s multiple comparison tests) were performed using Xlstat-Pro (win) 7.5.3. (Addinsoft, New York, NY, USA). Results were considered significant at *p* < 0.05 confidence level.

## 4. Conclusions

In this work, chitosan, chitosan–gelatine, and chitosan–gum arabic films without and with the addition of lemon balm extract were developed and characterized through physical, optical, barrier and antioxidant properties. The results indicate an improvement in the functional properties of the films containing *Melissa officinalis* extract, suggesting their potential for applications in active food packaging systems. However, these results should be further validated through applications, more specifically, studies on real food models and shelf-life evaluation.

All pH values of the polymer dispersions were in a range from 4.5 to 5, with some differences between neat CS (lower due to the acidity of the solvent) and polymer blends (higher, due to the dilution in water and because of creation of the bonds between cationic chitosan and anionic intrinsic properties of gelatine and gum arabic). The observed pH variations between polymers were attributed to differences in the intrinsic acidity/basic character of the mixtures and creation of polymer chains, while after the addition of the extract, changes were measurable only for the neat CS film, possibly due to the dilution effect of the aqueous extract.

CS–GA films showed the highest gas permeability, likely due to their less compact structure, while extract addition further increased permeability, particularly in blended systems, as a consequence of enhanced moisture content and water affinity within the matrix.

The improvements were attributed to the presence of phenolic compounds acting as active functional components within the polymer matrix. These compounds enhance antioxidant activity through hydrogen atom or electron donation, while also promoting intermolecular interactions (e.g., hydrogen bonding) with the polysaccharide/protein chains. It is possible that such interactions contribute to a more cohesive network structure, resulting in improved barrier properties and modified optical properties. Furthermore, the observed results suggest a clear relationship between the structure and film properties, not only depending on the polymer type but also with the addition of bioactive compounds that influence both the functional performance and physicochemical characteristics of the films.

Also, it is necessary to highlight the importance of green extraction as a promising and environmentally friendly technology to obtain plant extracts that can be incorporated into different products. Plant extracts contain a wide range of bioactive molecules with lots of benefits for food or cosmetic products, as well as for packaging materials.

## Figures and Tables

**Table 1:** Moisture content (*M*) determined in samples stored at 33% RH, solubility in water (*S*) and water absorption (*WA*) of biopolymer films.

Sample	*M* (%)	*S* (%)	*WA* (%)
CS	19.76 ± 0.70 ^b^	34.89 ± 0.84 ^a^	687.13 ± 217.98 ^a^
CS E	24.51 ± 2.43 ^a^	37.80 ± 3.37 ^a^	466.24 ± 144.22 ^a^
CS-GEL	13.19 ± 0.28 ^c^	38.20 ± 2.23 ^a^	987.22 ± 123.12 ^b^
CS-GEL E	14.26 ± 1.10 ^c^	39.63 ± 0.57 ^a^	1038.85 ± 15.67 ^b^
CS-GA	15.66 ± 0.19 ^c^	33.12 ± 4.15 ^a^	1398.74 ± 128.96 ^bc^
CS-GA E	14.22 ± 0.59 ^c^	36.69 ± 5.83 ^a^	1032.79 ± 186.97 ^b^

^a–c^ Different lowercase superscript letters within a column denote significant differences (*p* < 0.05) between values obtained for different biopolymer formulations.

**Table 2:** Colour parameters (*L*, *a*, *b*), total colour change and film transparency.

Sample	L*	a*	b*	Δ*E*	*T*_600_ (mm^−1^)
CS	89.77 ± 0.46 ^a^	−0.60 ± 0.35 ^b^	3.35 ± 1.53 ^c^	0.00 ± 0.00 ^c^	1.09 ± 0.11 ^ab^
CS E	72.66 ± 2.91 ^b^	0.37 ± 0.28 ^ab^	13.80 ± 1.87 ^b^	20.06 ± 3.45 ^b^	2.00 ± 0.20 ^a^
CS-GEL	90.46 ± 0.46 ^a^	−0.51 ± 0.14 ^b^	0.21 ± 1.59 ^c^	3.31 ± 1.65 ^c^	0.68 ± 0.07 ^b^
CS-GEL E	74.90 ± 0.69 ^b^	0.11 ± 0.39 ^b^	14.41 ± 0.63 ^b^	21.06 ± 0.91 ^b^	1.35 ± 0.13 ^ab^
CS-GA	89.88 ± 0.84 ^a^	−0.16 ± 0.94 ^b^	1.48 ± 3.66 ^c^	3.49 ± 2.36 ^c^	1.67 ± 0.17 ^ab^
CS-GA E	69.79 ± 3.92 ^c^	2.90 ± 1.66 ^a^	20.44 ± 4.95 ^a^	26.44 ± 6.29 ^a^	2.10 ± 0.21 ^a^

^a–c^ Different lowercase superscript letters within a column denote significant differences (*p* <0.05).

**Table 3:** Thickness (*l*) and water vapour barrier properties (*WVP* and *WVTR*) of films.

Sample	*l* (µm)	*WVP* (g m^−1^ s^−1^ Pa^−1^)∙10^−10^	*WVTR* (g m^−2^ s^−1^)∙10^−3^
CS	69.50 ± 20.02 ^c^	2.92 ± 0.31 ^a^	9.24 ± 0.99 ^ab^
CS E	73.00 ± 17.22 ^bc^	3.23 ± 0.33 ^a^	10.20 ± 1.05 ^a^
CS-GEL	120.50 ± 37.71 ^a^	2.31 ± 0.24 ^b^	7.32 ± 0.76 ^ab^
CS-GEL E	107.50 ± 10.71 ^ab^	2.39 ± 0.07 ^b^	7.58 ± 0.20 ^ab^
CS-GA	70.60 ± 17.87 ^c^	2.17 ± 1.26 ^abc^	6.98 ± 0.50 ^b^
CS-GA E	112.75 ± 10.24 ^a^	2.14 ± 0.04 ^c^	6.79 ± 0.10 ^ab^

^a–c^ Different lowercase superscript letters within a column denote significant differences (*p* < 0.05) between values obtained for different biopolymer formulations.

**Table 4:** Antioxidant activity of films.

Sample	DPPH Inhibition (%)	FRAP (mg AAE/g Film)
CS	14.71 ± 4.12 ^de^	6.93 ± 2.68 ^b^
CS E	59.06 ± 3.35 ^a^	44.05 ± 10.94 ^a^
CS-GEL	8.16 ± 2.35 ^e^	5.33 ± 1.23 ^b^
CS-GEL E	29.49 ± 2.35 ^bc^	36.90 ± 6.46 ^a^
CS-GA	23.82 ± 0.39 ^cd^	8.57 ± 1.06 ^b^
CS-GA E	37.92 ± 2.16 ^b^	41.23 ± 1.06 ^a^

^a–e^ Different lowercase superscript letters within a column denote significant differences (*p* < 0.05) between values obtained for different biopolymer formulations.

**Table 5:** Formulation of biopolymer films.

Abbreviation	Chitosan (CS) (%, *w*/*v*)	Gelatine (GEL) (%, *w*/*v*)	Gum Arabic (GA) (%, *w*/*pdm*)	Glycerol (%, *w*/*pdm*)	Lemon Balm Extract (%, *v*/*v*)
CS	2	0	0	0.4	0
CS E	2	0	0	0.4	10
CS-GEL	2	1	0	0.6	0
CS-GEL E	2	1	0	0.6	10
CS-GA	2	0	10	0.2	0
CS-GA E	2	0	10	0.2	10

## Data Availability

All of the data are contained in the present article.
